# Alteration of TLR3 pathways by glucocorticoids may be responsible for immunosusceptibility of human corneal epithelial cells to viral infections

**Published:** 2009-05-08

**Authors:** Yuko Hara, Atsushi Shiraishi, Takeshi Kobayashi, Yuko Kadota, Yuji Shirakata, Koji Hashimoto, Yuichi Ohashi

**Affiliations:** 1 Department of Ophthalmology, Ehime University School of Medicine, Shitsukawa, Japan; 2 Department of Ophthalmology and Regenerative Medicine, Ehime University School of Medicine, Shitsukawa, Japan; 3 Department of Dermatology, Ehime University School of Medicine, Shitsukawa, Japan

## Abstract

Purpose: The toll-like receptor 3 (TLR3) recognizes viral double-stranded RNA and its synthetic analog polyriboinosinic-polyribocytidylic acid (poly(I:C)), and the activation of TLR3 is known to induce the production of type I interferon (IFN) and inflammatory cytokines/chemokines. The purpose of this study was to determine the role played by innate responses to a herpes simplex virus 1 (HSV-1) infection of the corneal epithelial cells. In addition, we determined the effects of immunosuppressive drugs on the innate responses.

Methods: Cultured human corneal epithelial cells (HCECs) were exposed to poly(I:C), and the expressions of the mRNAs of the cytokines/chemokines macrophage-inflammatory protein 1 alpha (*MIP1-α*), macrophage-inflammatory protein 1 beta (*MIP1-β*), interleukin-6 (*IL-6*), interleukin-8 (*IL-8*), regulated on activation, normal T cell expressed and secreted (*RANTES*), Interferon-beta (*IFN-β*), and *TLR3* were determined using real-time reverse transcription-polymerase chain reaction (RT-PCR). The effects of dexamethasone (DEX, 10^-6^ or 10^-5^ M) and cyclosporine A (CsA, 10^-6^ or 10^-5^ M) on the expression of these cytokines and *TLR3* were also determined using real-time RT-PCR. Levels of MIP1-α, MIP1-β, IL-6, IL-8, RANTES, and IFN-β were measured using the enzyme-linked immunosorbent assay (ELISA). The activation of nuclear factor kappa B (NFκB) and interferon regulatory factor 3 (IRF3) in HCECs was assessed by immunohistochemical staining. The effects of DEX and CsA on HCECs exposed to HSV-1 (McKrae strain) were also examined.

Results: The expressions of *MIP1-α*, *MIP1-β*, *IL-6*, *IL-8*, *RANTES*, *IFN-β*, and *TLR3* were up-regulated in HCECs exposed to poly(I:C). The poly(I:C)-induced expressions of *IL-6* and *IL-8* were down-regulated by both DEX and CsA, while the expressions of *IFN-β* and *TLR3* were suppressed by DEX alone. Similarly, the poly(I:C)-induced activation of NFκB was decreased by both DEX and CsA, and the activation of IRF3 was reduced by DEX alone. When HCECs were inoculated with HSV-1, DEX led to a decrease in the expression of *IL6*, *IFN-β*, and *TLR3*, and an extension of plaque formation.

Conclusion: These results indicate that DEX may increase the susceptibility of HCECs to viral infections by altering the TLR3 signaling pathways.

## Introduction

The toll-like receptors (TLRs) are a family of innate immune receptors that recognize the conserved structures of microbes, termed pathogen-associated molecular patterns (PAMPs). The TLR system has been extensively studied in immune cells, e.g. in macrophages, and recent studies have demonstrated that epithelial cells also express TLRs. Thus, respiratory epithelial cells express TLR 1–10 [1,2], epidermal keratinocytes express TLR1, 2, 4, and 5 [3,4], intestinal epithelial cells express TLR1–4, 6, and 9 [5], and female reproductive tract epithelial cells express TLR1–9 [6]. In the eye, human corneal epithelial cells express TLR 1–7, 9, and 10 [7], and human conjunctival epithelial cells express TLR 1–6 and 9 [8].

The question then arises whether the TLRs play a role in the keratitis caused by the herpes simplex virus (HSV). It is known that treatment of stromal keratitis with topical acyclovir significantly reduces the number of patients who suffer serious visual impairment. However, keratitis often recurs in immunocompromised hosts or in individuals who receive steroid therapy for a long period of time. In fact, topical or systemic application of glucocorticoids results in the reactivation of herpes keratitis [9,10], and glucocorticoids are contraindicated for epithelial keratitis because they can worsen the clinical course to virus-induced geographic keratitis [11].

Recent studies have shown that a TLR3 ligand, which is a double-stranded RNA (dsRNA) can activate different types of epithelial cells, e.g. airway epithelial cells, female reproductive tract epithelial cells, and corneal epithelia cells [7,12,13]. TLR3 is the only TLR that does not interact with myeloid differentiation factor 88 (MyD88) as a signaling adaptor [14]. TLR3 interacts directly with the adaptor protein, Toll/interleukin-1 receptor (TIR) domain-containing adaptor inducing IFN-β (TRIF), which is also called the TIR-containing adaptor molecule (TICAM-1). TRIF/TICAM-1 activates the transcription factor NFκB and the interferon regulatory factor 3 (IRF3) [15,16]. The activation of NFκB leads to the production of inflammatory cytokines/chemokines, and the activation of IRF3 elicits anti-viral responses, especially through the production of type I IFN [15,17,18]. The production of type I IFN is the first line of defense against viral infections, and it acts by limiting the early replication of viruses [19,20]. Deonarain et al. [21] demonstrated that IFN-β is crucial for this process, because IFN-β-deficient mice are highly susceptible to viral infections.

TLR3 recognizes dsRNA and would not be expected to detect DNA from a DNA virus, such as HSV. However, it is known that most viruses synthesize dsRNA during their replication [22], and therefore TLR3 should be able to recognize HSV. Recently, Kariko et al. [23] reported that TLR3 is stimulated by cellular mRNA, and Ashkar et al. [24] reported that the delivery of ligands for TLR3, but not TLR4, protected against HSV-2 infections. Hayashi et al. [25] reported that herpes simplex virus 1 (HSV-1) elicited inflammatory cytokines via TLR3 and TLR9 in the corneal epithelial cells. Thus, corneal epithelial cells may play a role as the first line of defense against viral infection, including HSV infection, through the TLRs.

The purpose of this study was to determine the role played by innate responses in controlling HSV-1 infection of the corneal epithelial cells. In addition, we examined whether immunosuppressive drugs altered the HSV-1 infection of the cornea. We shall show that polyriboinosinic-polyribocytidylic acid (poly(I:C)), a TLR3 agonist, can induce anti-viral responses in corneal epithelial cells. However, these anti-viral responses can be altered by dexamethasone (DEX) and cyclosporine A (CsA).

## Methods

### Human subjects

All procedures on human subjects conformed to the tenets of the Declaration of Helsinki [26]. The experimental protocol for these experiments was approved by the Institutional Review Board of Ehime University.

### Chemicals and cell cultures

All reagents used for the cell cultures were purchased from Invirogen (Carlsbad, CA). Primary human corneal epithelial cells (HCECs) were isolated from human corneoscleral buttons dissected from eyes acquired from an American Eye Bank(Sight Life Seattle WA) as reported [27]. Briefly, the buttons were carefully denuded of the endothelial cells and adherent iris. After digestion with 1.2 U/ml dispase at 4 ºC for 24 h, the loosened epithelial sheets were removed and dispersed into single cells by enzyme digestion with 0.1% trypsin and 0.02% EDTA. Then, the HCECs were cultured in serum-free modified MCDB 153 type II medium, supplemented with insulin (5 μg/ml), hydrocortisone (5×10^-7^ M), ethanolamine (0.1 mM), phosphoethanolamine (0.1 mM), Insulin-like growth factor-1 (IFG-1; 10 ng/ml), Epidermal growth factor (EGF; 0.1 ng/ml), and Ca^2+^ (0.06 mM). The medium was changed every 2 days.

To determine the effects of DEX and CsA on the poly(I:C)-induced expression of  cytokines/chemokines, HCECs were cultured with hydrocortisone-free, modified MCDB 153 type II medium for 24 h, then incubated with 100 ng/ml of poly(I:C) in the presence or absence of DEX (10^-6^ or 10^-5^ M) or CsA (10^-6^ or 10^-5^ M). In the CsA control, CsA was substituted with 0.01% dimethyl sulfoxide (DMSO), which was also used to reconstitute the CsA. After 24 h of stimulation the cells and supernatants were collected.

### Real-time PCR analysis

Total RNA was extracted from the cultured HCECs using RNeasy kit (Qiagen, Valencia, CA), and then reverse-transcribed using Omniscript Reverse Transcriptase (Qiagen) according to the manufacturer’s protocols. Real-time PCR was performed with the DyNAmo SYBR Green qPCR Kit (Finnzymes, Espoo, Finland) as follows: 95 ºC for 15 min; 40 cycles of denaturation at 95 ºC for 10 s; annealing at 60 ºC for 20 s; and extension at 72 ºC for 30 s using the OPticon 2 DNA Engine (BioRad, Hercules, CA). The primer pairs used for real-time PCR are listed in Table 1. The C_t_ values were determined by the Opticon 2 software, and the amount of each mRNA was calculated relative to the amount of Glyceraldehyde 3 phosphate dehydrogenase (*GAPDH*) mRNA in the same samples [28]. Each run was completed with a melting curve analysis to confirm the specificity of the amplification and the absence of primer dimers.

**Table 1:** Primer pairs for real-time PCR.

**Gene**	**Forward primer**	**Reverse primer**	**Product size (bp:Accession number)**
*IL-6*	TACCCCCAGGAGAAGATTCC	TTTTCTGCCAGTGCCTCTTT	175 : M29150
*IL-8*	GTGCAGTTTTGCCAAGGAGT	CTCTGCACCCAGTTTTCCTT	196 : BC013615
*MIP-1α*	TGCAACCAGTTCTCTGCATC	TTTCTGGACCCACTCCTCAC	198 : BC071834
*MIP-1β*	AAGCTCTGCGTGACTGTCCT	GCTTGCTTCTTTTGGTTTGG	211 : NM_002984
*IFN-β*	CATTACCTGAAGGCCAAGGA	CAGCATCTGCTGGTTGAAGA	178 : V00534
*RANTES*	GAGGCTTCCCCTCACTATCC	CTCAAGTGATCCACCCACCT	155 : BC008600
*TLR3*	AGCCTTCAACGACTGATGCT	TTTCCAGAGCCGTGCTAAGT	201 : NM_003265
*G3PDH*	CGACCACTTTGTCAAGCTCA	AGGGGAGATTCAGTGTGGTG	203 : BT006893

### Measurement of proinflammatory cytokines/chemokines production

The concentrations of MIP1-α, MIP1-β, IL-6, IL-8, RANTES, and IFN-β in the supernatants of the cultured HCECs were determined using an ELISA kit (R&D Systems, Minneapolis, MN) following the manufacturer’s protocols.

### Immunostaining for NFκB and IRF3

HCECs were cultured on CultureSlides (BD Falcon, Bedford, MA) with 100 ng/ml of poly(I:C) in the presence or absence of DEX (10^-5^ M) or CsA (10^-5^ M) for 3 h. Cells were washed three times with phosphate-buffered saline (PBS), then fixed for 15 min in 3.2% paraformaldehyde (PFA)/PB. After washing with PBS, cells were permeabilized with 0.1% Triton X-100 for 5 min, followed by incubation with primary antibodies to NFκB p65 (0.2 μg/ml; Santa Cruz Biotechnology, Santa Cruz, CA) or to IRF3 (0.2 μg/ml; Santa Cruz Biotechnology) in 1% bovine serum albumin (BSA)/PBS at 4 ºC for 16 h. After washing with PBS, the slides were incubated with specific secondary antibodies, then incubated with appropriate fluorescein (FITC) conjugated antibodies (Pierce, Rockford, IL). Finally, the slides were coverslipped using an anti-fading mounting medium (Vector, Burlingame, CA). For the controls, sections were treated with normal rabbit immunoglobulin G (IgG), and no positive staining was detected with any of the antibodies.

### Herpes simplex virus 1 (HSV-1) infection

Stocks of the McKrae strain of HSV-1 were propagated on African green monkey kidney (Vero) cells grown in complete Dulbecco’s modified Eagle’s medium (DMEM) containing 10% fetal bovine serum (FBS), 1% penicillin, and streptomycin. The titer of virus stocks was determined by the standard plaque assay on Vero cells, and titers were expressed as plaque-forming units (PFU)/ml. Stocks were stored at –70 ºC in 1 ml aliquots, and a fresh aliquot of stock virus was used for each experiment.

HCECs were cultured in a hydrocortisone-free, modified MCDB 153 type II medium for 24 h, and cultured in the presence or absence of DEX (10^-5^ M) or CsA (10^-5^ M) prior to exposure to HSV-1. For the plaque assay, HCECs were inoculated with HSV-1 at a multiplicity of infection (MOI) of 50 for 48 h, and the cells were then fixed with 10% formalin and stained with crystal violet. The area of the plaques was measured by Adobe Photoshop software (Adobe Systems Incorporated, San Jose, CA) to evaluate the efficiency of infection. The supernatants were also collected to evaluate the concentration of HSV-1 DNA by real-time PCR. To examine the participation of the TLR3 systems in signaling the HSV-1 infection on HCECs, the HCECs were pre-incubated with or without DEX, and then inoculated with HSV-1. To collect the cells before plaque formation, the time period from inoculation to testing was reduced to 24 h, and the inoculated dose increased to a MOI of 1,000, to allow detection of changes in inflammatory cytokines/chemokines. Therefore, HCECs were pre-incubated with or without DEX (10^-5^ M), followed by HSV-1 inoculation with a MOI of 1,000, and the cells collected for real-time PCR after 24 h.

### Statistical analyses

Each experiment was repeated 3 times, and representative results are shown in the figures. Values are presented as means±standard deviations (SDs). Differences between the groups were determined by two-tailed paired t-tests. A p-value of <0.05 was considered to be statistically significant.

## Results

### Poly(I:C)-induced TLR3 signaling pathway

**Figure 1:**
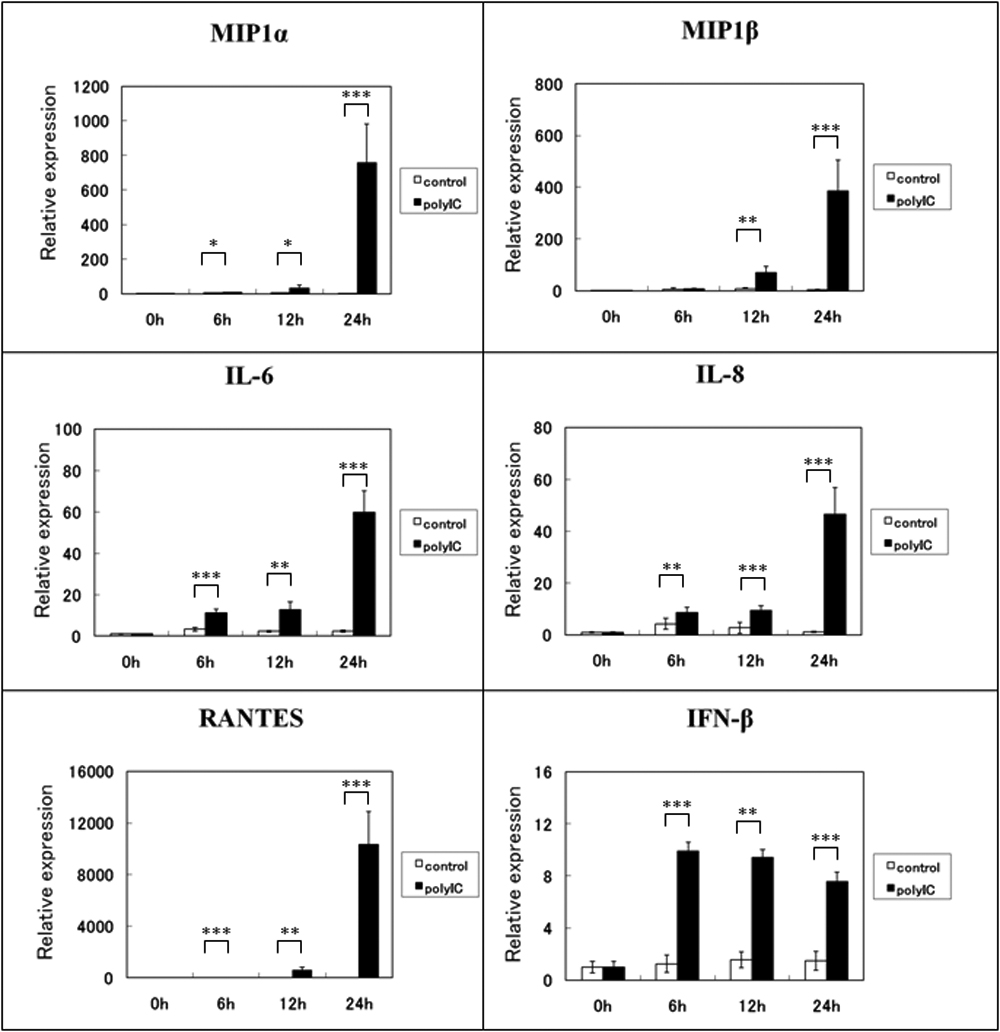
Expression of the mRNAs of cytokines and chemokines by HCEs exposed to poly(I:C), a TLR3 ligand. Total RNA was isolated from HCECs at 6, 12, and 24 h after poly(I:C) exposure, and the expressions of the mRNAs of *MIP1-α*, *MIP1-β*, *IL-6*, *IL-8*, *RANTES*, and *IFN-β* were determined by real-time PCR. The relative level of expression of each cytokine and chemokine mRNA is normalized to the level of *G3PDH* mRNA expression. The p values were calculated using two-tailed paired t-tests, (*p<0.05, **p<0.01, ***p<0.001).

**Figure 2:**
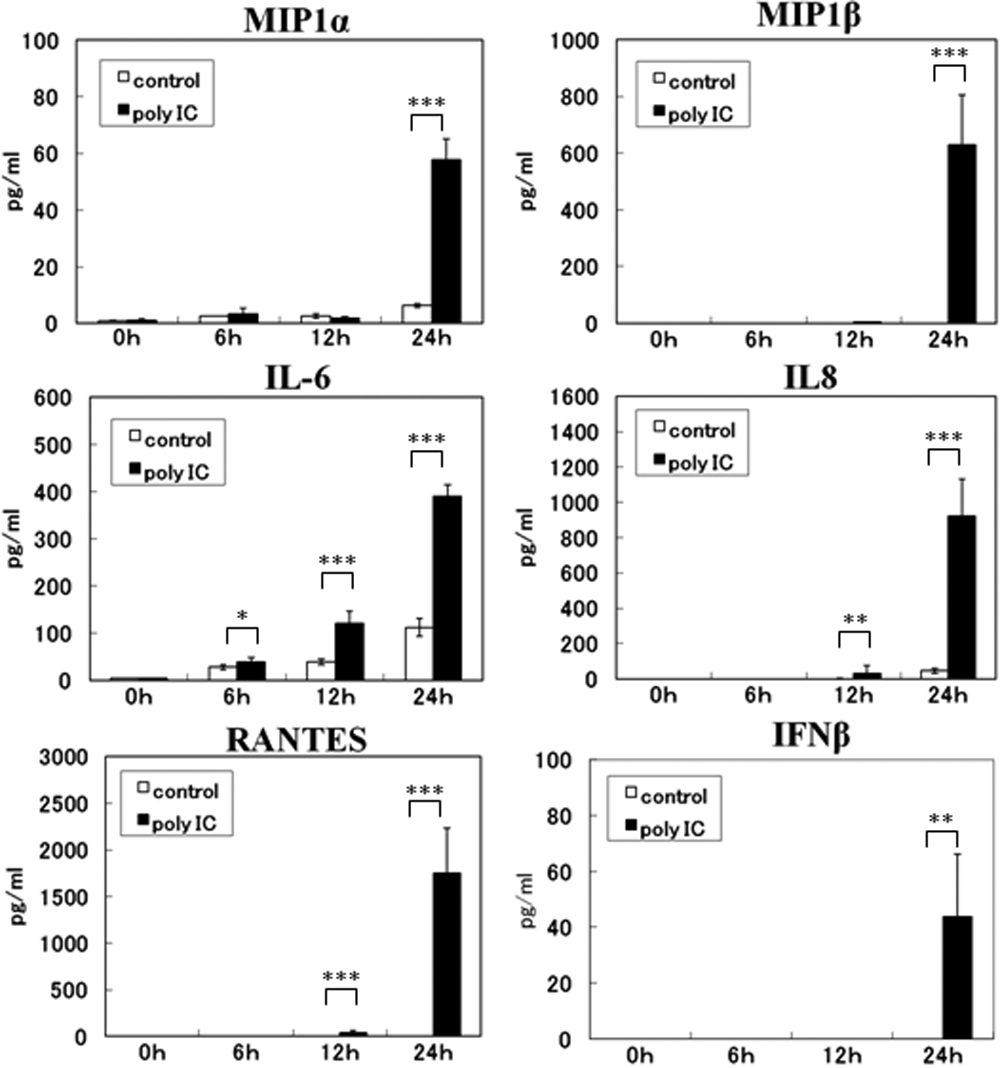
Cytokines and chemokines secreted by HCECs treated with poly(I:C). Culture medium was collected at 6, 12, and 24 h after poly(I:C) stimulation and analyzed for MIP1-α, MIP1-β, IL-6, IL-8, RANTES, and IFN-β protein by ELISA. The p values were calculated using two-tailed paired t-tests (*p<0.05, **p<0.01, ***p<0.001).

To determine whether the TLR3/TRIF pathway is active in cultured HCECs, the HCECs were incubated with 100 ng/ml of poly(I:C) for 6, 12, and 24 h. Real time RT-PCR was then performed on the cells with primer pairs for *MIP1-α*, *MIP1-β*, *IL-6*, *IL-8*, *RANTES*, *IFN-β*, and *TLR3*. After stimulation by poly(I:C), the expression of the mRNA of *MIP1-α*, *IL-6*, *IL-8*, and *RANTES* were up-regulated as early as 6 h, and the level had increased 750 fold, 60 fold, 50 fold, and 10,000 fold, respectively, at 24 h. *MIP1-β* was also up-regulated at 12 h and reached about 400 fold at 24 h. *IFN-β* was up-regulated 9.9 fold within 6 h, which was maintained for 24 h (Figure 1). *TLR3* was also up-regulated at 12 h, and the level had increased about 40 fold after 24 h (Figure 2A). The expressions of inflammatory cytokines/chemokines and *TLR3* were not significantly altered without poly(I:C) stimulation (Figure 1 and Figure 2A).

**Figure 3:**
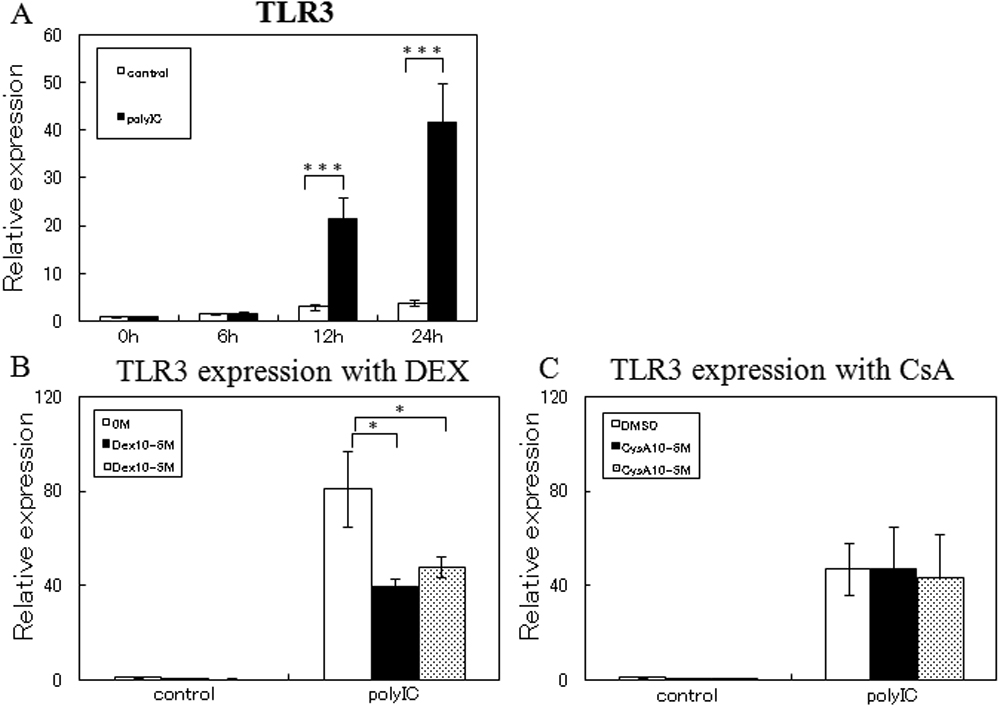
Effect of DEX and CsA on the expression of TLR3 by HCECs exposed to poly(I:C). Total RNA was isolated from HCECs at 6, 12, and 24 h after poly(I:C) stimulation (**A**), or from HCECs cultured with or without of DEX (**B**) or CsA (**C**) for 24 h and stimulated with poly(I:C) for 24 h. The expression of the mRNA of *TLR3* was determined by real-time PCR. The relative level of expression of each cytokine and chemokine mRNA is normalized against *G3PDH* mRNA expression. The p values were calculated using two-tailed paired t-tests (*p<0.05, **p<0.01, ***p<0.001).

The supernatants of the culture media were collected at 0, 6, 12, and 24 h, and the levels of MIP1-α, MIP1-β, IL-6, IL-8, RANTES, and IFN-β was evaluated using ELISA. The levels of MIP1-α, MIP1-β, and RANTES in the supernatant were elevated from undetectable levels at 0 h to 57.6 pg/ml, 630 pg/ml, and 1748.7 pg/ml, respectively, at 24 h after poly(I:C) stimulation. There was a slight but not significant elevation without poly(I:C) stimulation. The levels of IL-6 and IL-8 were slightly elevated without poly(I:C) stimulation, but were significantly elevated to 390 pg/ml and 920 pg/ml, respectively, at 24 h after poly(I:C) stimulation. The level of IFN-β was elevated to 43.8 pg/ml by poly(I:C) after 24 h, and no production of IFN-β was found without poly(I:C) stimulation (Figure 3).

### Effect of DEX and CsA on TLR 3 signaling pathway

To determine whether DEX and CsA altered the expressions of the poly(I:C)-induced TLR3 and inflammatory cytokines/chemokines, HCECs were cultured with 100 ng/ml of poly(I:C) with or without DEX (10^-6^ or 10^-5^ M) or CsA (10^-6^ or 10^-5^ M). After 24 h, the cells and supernatants were collected, and the expression of the mRNAs and proteins of IL-6, IL-8, IFN-β, and TLR3 were evaluated by real-time PCR and ELISA.

Incubation with DEX down-regulated the poly(I:C)-induced expression of *TLR3* mRNA about 0.5 fold with 10^-6^ M and 0.6 fold with 10^-5^ M of DEX, whereas no effect was found when incubated with CsA (Figure 2B,C).

**Figure 4:**
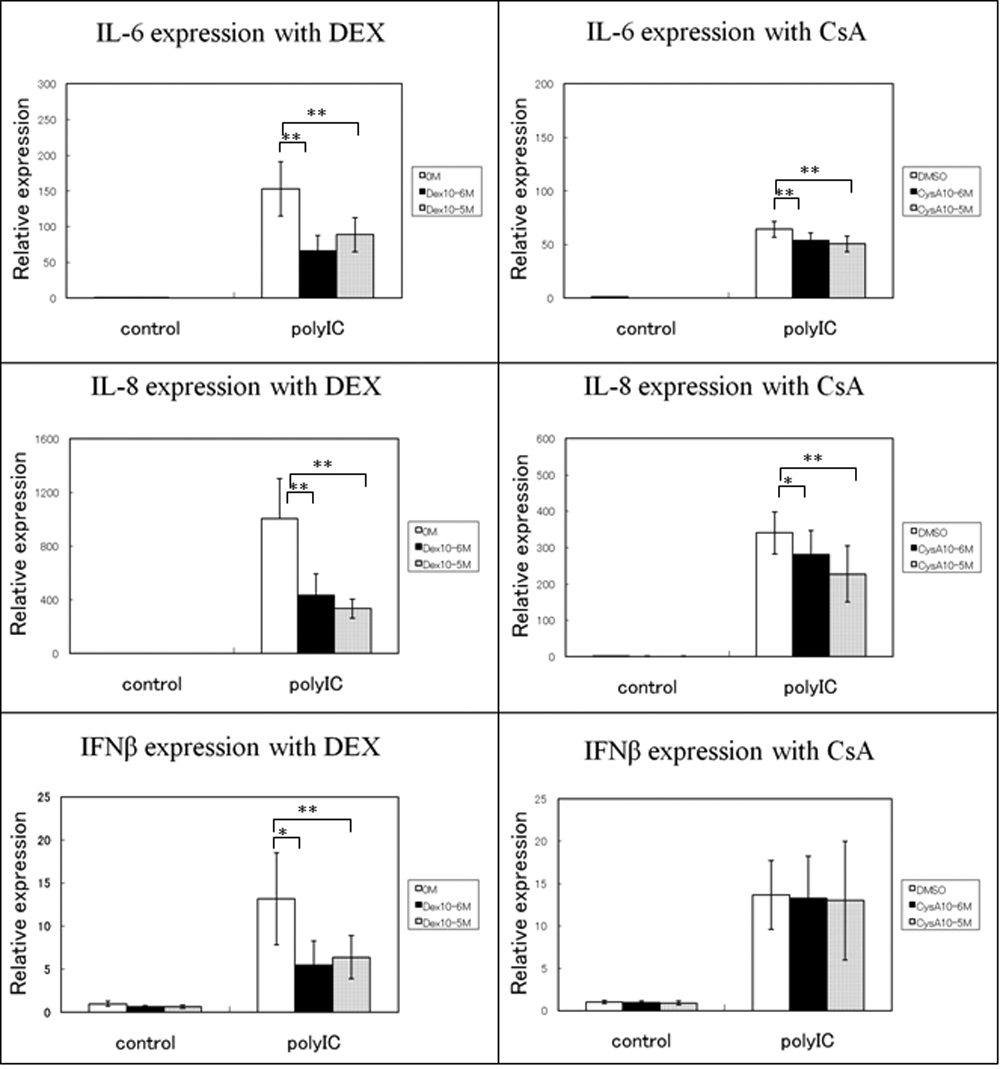
Effect of DEX and CsA on the expression of cytokines and chemokines by HCEs treated with poly(I:C). Total RNA was isolated from HCECs cultured with or without of DEX or CsA for 24 h and stimulated with poly(I:C) for 24 h. The expressions of the mRNAs of *IL-6*, *IL-8*, and *IFN-β* were determined by real-time PCR. The relative level of expression of each cytokine and chemokine mRNA is normalized to the level of *G3PDH* mRNA expression. The p values were calculated using two-tailed paired-tests (*p<0.05, **p<0.01, ***p<0.001).

**Figure 5:**
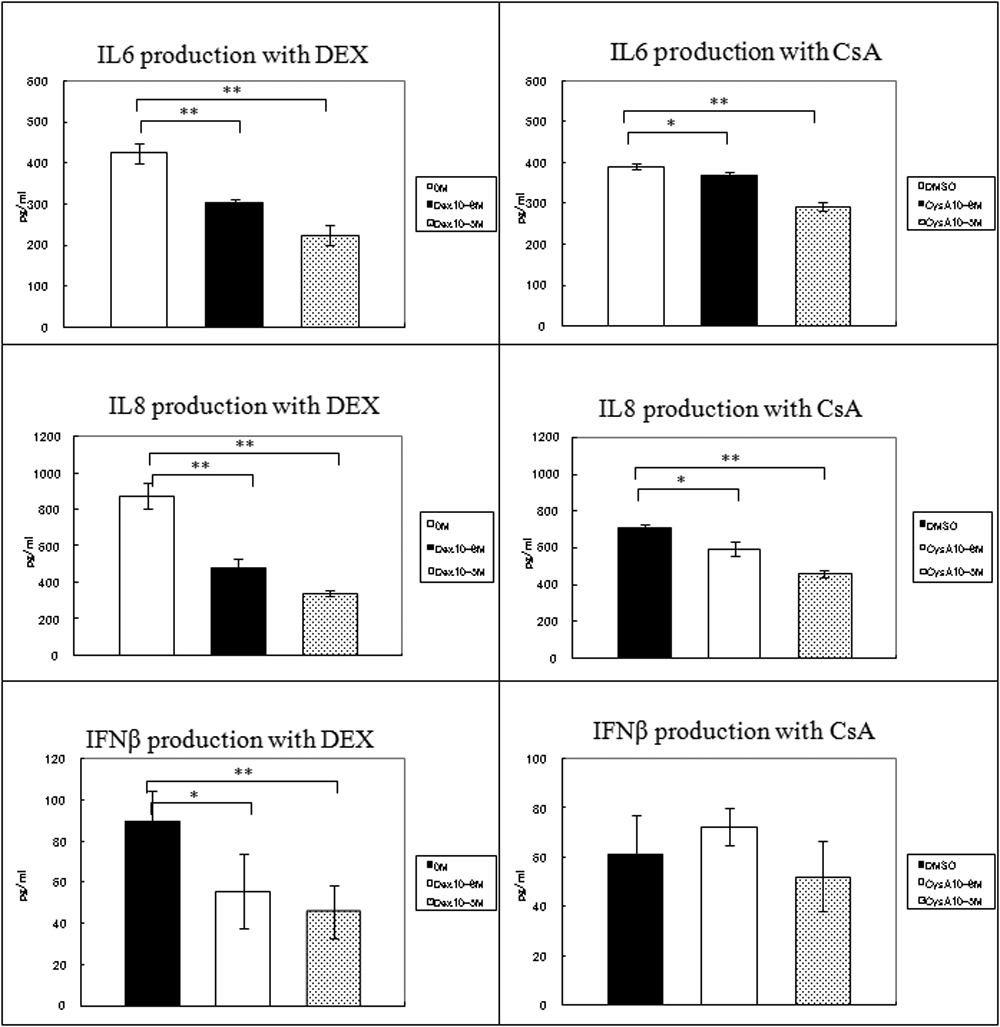
Cytokines and chemokines secreted by HCECs stimulated with poly(I:C) and cultured with or without DEX or CsA for 24 h. Culture medium was collected 24 hours after poly(I:C) stimulation and analyzed for the presence of IL-6, IL-8, and IFN-β protein by ELISA. The p values were calculated using two-tailed paired-tests, (*p<0.05, **p<0.01, ***p<0.001).

Incubation with DEX down-regulated the poly(I:C)-induced expression of the mRNA of *IL-6* about 0.4 fold with 10^-6^ M and 0.5 fold with 10^-5^ M of DEX (Figure 4). ELISA also showed that the poly(I:C) induced IL-6 production was decreased about 0.6 fold with 10^-6^ M and 0.5 fold with 10^-5^ M of DEX (Figure 5). The poly(I:C)-induced expressions of the mRNA and proteins of IL-8 were more significantly down-regulated by DEX, and the decrease was dose-dependent. Real-time PCR showed that the expression of the mRNA of *IL-8* was down-regulated about 0.4 fold with 10^-6^ M and 0.3 fold with 10^-5^ M of DEX. ELISA also showed a reduced production of IL-8 protein of about 0.5 fold with 10^-6^ M and 0.4 fold with 10^-5^ M of DEX (Figure 4 and Figure 5). DEX also down-regulated the poly(I:C)-induced mRNA expression of *IFN-β* by about 0.5 fold with 10^-6^ M and 10^-5^ M of DEX and decreased IFN-β production by about 0.6 fold with 10^-6^ M and 0.5 fold with 10^-5^ M of DEX (Figure 4 and Figure 5).

The effect of CsA on the poly(I:C)-induced inflammatory cytokine/chemokine expression was not as extensive as with DEX. However, the poly(I:C)-induced *IL-6* mRNA expression was down-regulated about 0.8 fold with 10^-5^ M of CsA, and ELISA showed that the poly(I:C) induced IL-6 production was reduced about 0.7 fold with 10^-5^ M of CsA (Figure 4 and Figure 5). The poly(I:C)-induced *IL-8* mRNA expression was also down-regulated about 0.65 fold with 10^-5^ M of CsA (Figure 4), and ELISA showed a decrease in production of about 0.65 fold with 10^-5^ M of CsA (Figure 5). Interestingly, CsA had no effect on poly(I:C)-induced *IFN-β* mRNA expression or production (Figure 4 and Figure 5).

### Immunohistochemical staining for NFκB and IRF3

**Figure 6:**
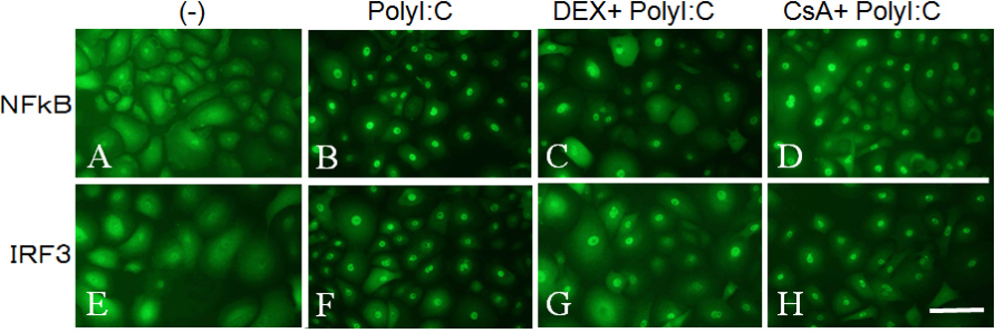
Immunohistochemical staining for NFκB and IRF3 in HCECs stimulated with poly(I:C) and cultured with or without of DEX or CsA for 24 h. NFκB p65 staining without poly(I:C, **A**), with poly(I:C, **B**), with DEX 10^-5^M and poly(I:C, **C**), and with CsA 10^-5^M and poly(I:C, **D**). IRF3 staining without poly(I:C, **E**), with poly(I:C, **F**), with DEX 10-5M and poly(I:C, **G**), and with CsA 10-5M and poly(I:C, **H**). Scale bar, 100 µm. Activated NFκB p65 and IRF-3 were clearly detected in the nuclei of most of cultured HCECs 3 h after stimulation by poly(I:C, **B** and **F**). In the presence of DEX, NFκB p65 and IRF-3 were detected in the nuclei of some HCECs but only in the cytosol of other HCECs (**C**, **G**). In the presence of CsA, NFκB p65 staining was detected in more HCEC nuclei after exposure to CsA than to DEX (D), while IRF3 was detected only in the nuclei of cultured HCECs (**H**).

The effect of DEX (10^-5^ M) or CsA (10^-5^ M) on the activation of NFκB and IRF-3 was determined immunohistochemically after 3 h of stimulation by poly(I:C). NFκB p65 and IRF-3 staining were weakly detected in the cytosol of cultured HCECs without poly(I:C) stimulation (Figure 6A,E), but activated NFκB p65 and IRF-3 were clearly detected in the nuclei of most of cultured HCECs 3 h after stimulation by poly(I:C; Figure 6B,F). After stimulation by poly(I:C) in the presence of DEX, NFκB p65 and IRF-3 were detected in the nuclei of some HCECs but only in the cytosol of other HCECs (Figure 6C,G). After stimulation by poly(I:C), NFκB p65 staining was detected in more HCEC nuclei after exposure to CsA than to DEX, but some HCECs were stained only in the cytosol when exposed to CsA (Figure 6D). IRF3 was detected only in the nuclei of cultured HCECs after 3 h of stimulation by poly(I:C) in the presence of CsA (Figure 6H).

### Effect of DEX and CsA on Herpes simplex virus 1 (HSV-1) infection

**Figure 7:**
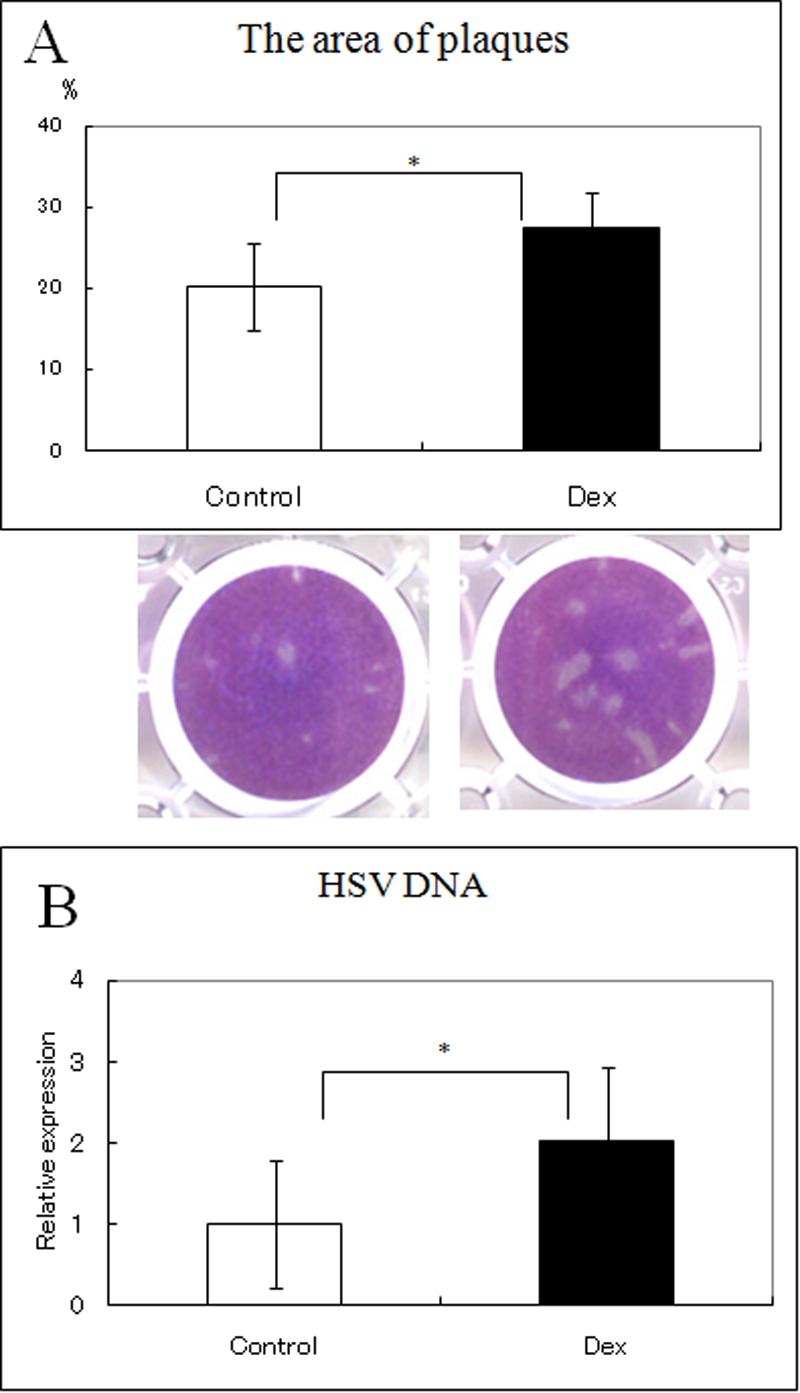
Effect of DEX and CsA on Herpes simplex virus 1 (HSV-1) infection. HCECs were cultured in the presence or absence of DEX (10^-5^ M), and inoculated with 50 MOI of HSV-1 for 48 h. The plaque area was increased when HCECs were pre-incubated with DEX (**A**). Real-time PCR results show a significantly higher level of *HSV-1* DNA in the supernatant with DEX (**B**). (*p<0.05)

To determine whether DEX and CsA affected the HSV-1 infection of HCECs, HCECs were cultured in the presence or absence of DEX (10^-5^ M) or CsA (10^-5^ M), and inoculated with HSV-1 at a MOI of 50. The plaque area was increased when HCECs were pre-incubated with DEX, but CsA had no effect on HSV-1 infection (Figure 7A). Real time PCR showed more *HSV-1* DNA in the supernatant of DEX-exposed HCECs (Figure 7B).

**Figure 8:**
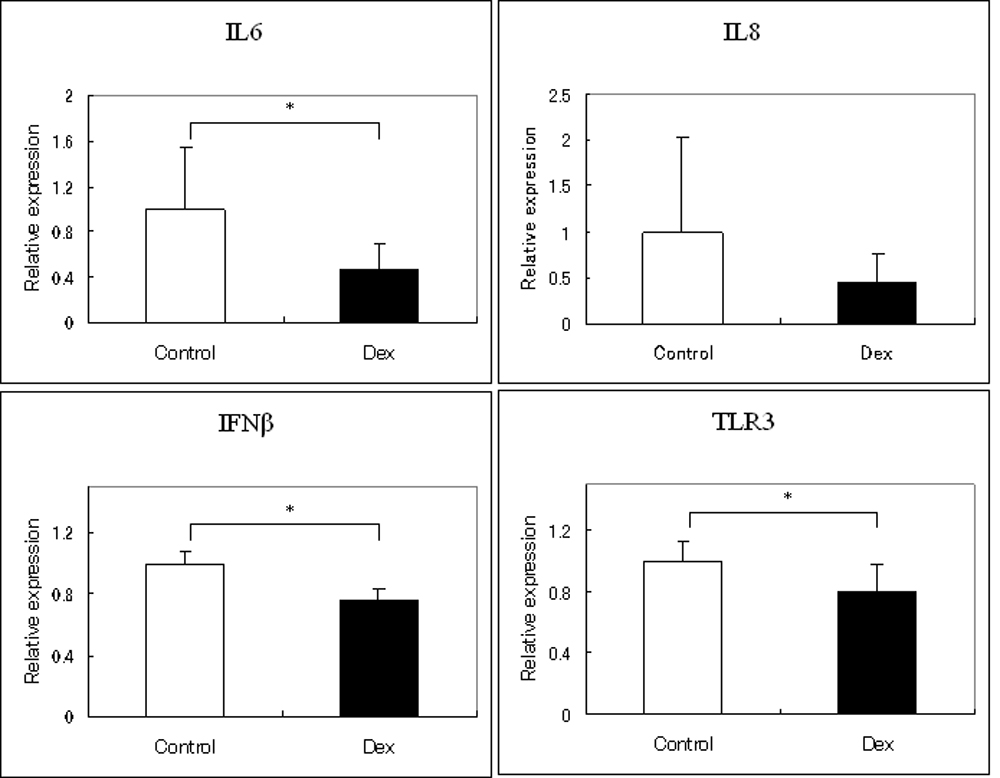
Effect of DEX and CsA on involvement of TLR3 signaling systems in HSV-1 infection of HCECs. HCECs were cultured in the presence or absence of DEX (10^-5^ M) and inoculated with 1,000 MOI of HSV-1 for 24 h. Real-time PCR shows that *IFN-β* and *TLR3* expression is down-regulated by DEX. *IL-6* and *IL-8* are also down-regulated, although the decrease of *IL-8* was not statistically significant. (*p<0.05)

In addition, we investigated the involvement of TLR3 signaling systems in HSV-1 infection of HCECs. Real-time PCR showed that the expressions of *IL6*, *IFN-β*, and *TLR3* were down-regulated by DEX when HCECs were inoculated with HSV-1 (Figure 8). *IL-6* and *IL-8* were also down-regulated, although the decrease was not statistically significant for *IL-8* (Figure 8).

## Discussion

Our results showed that poly(I:C), a TLR3 agonist, up-regulated the production of inflammatory cytokines/chemokines such as MIP1-α, MIP1-β, RANTES, IL-6, and IL-8, by activating NFκB.   Incubation of HCECs with poly(I:C) also activated IRF3 followed by IFN-β production. The up-regulated expression of TLR 3 by poly(I:C) indicates that the TLR3/TRIF signaling pathways were most likely activated by poly(I:C) in HCECs. This is consistent with previous reports [1,15-17]. The cytokines and chemokines investigated are known to have powerful effects in recruiting immune cells and stimulating the maturation of dendritic cells [29-31]. Therefore, we suggest that corneal epithelial cells, when the TLR3s are activated de novo, are able to recruit and activate immune cells against viral infections.

Our results showed that DEX and CsA inhibit the poly(I:C)-induced NFκB activation and the subsequent production of inflammatory cytokines/chemokines. Earlier studies have shown that the concentration of topically applied reagents in tears sharply decreases to less than 1/100 of the original concentration by one hour after administration, and keeps decreasing until only trace levels remain [32,33]. The concentrations of DEX and CsA used in this study were 1/500 and 1/5,000 of the concentration used in eye drops in a clinical setting (0.05%), and so the results should be clinically applicable.

Glucocorticoids, potent inhibitors of immune responses, act through glucocorticoid receptors (GRs) to depress the activities of other DNA-bound transcription factors, such as activator protein 1(AP-1) and NFκB [34-37]. CsA is known to inhibit T cell activation and proliferation [38]. Recent studies have shown that the inhibitory effects of CsA result from interference in the degradation of inhibitory kappaB (IκB) and a reduction in the transcriptional activity of the classic NFκB signaling pathway [39,40]. Our immunohistochemical results showed that DEX and CsA inhibit the poly(I:C)-induced nuclear translocation of NFκB, and these findings are in accord with earlier reports. Thus, the inhibition of inflammatory cytokines/chemokines by DEX and CsA in HCECs may result from the inhibition of NFκB, and this may be one of the mechanisms responsible for the immunosuppressive property of DEX and CsA.

DEX and CsA have different effects on the activation of IRF3 and IFN-β production, and both are part of the TRIF/TICAM-1 TLR3 signaling pathways [15,17,18]. DEX inhibited the poly(I:C)-induced IRF3 activation and the subsequent IFN-β production, while CsA inhibited neither IRF3 activation nor IFN-β production. The exact mechanism of action of DEX and CsA on IRF3 has still not been determined, however Reily et al. [41] have identified the glucocorticoid receptor-interacting protein 1 (GRIP1) to be an IRF3-interacting protein that facilitates IRF3-mediated transcription. They showed that the GRIP1:IRF3 interaction is blocked by the activation of GRs [41]. Our finding that DEX inhibited the poly(I:C)-induced IRF3 activation in HCECs is in accord with their findings.

The different effects of DEX and CsA on the activation of IRF3 and IFN-β production might also be explained by their differing effects on the expression of TLR3. Because the IFN-responsive element (ISRE) is located on the human TLR3 promoter region, it has been suggested that IFNα/β induces the expression of TLR3 [42,43]. It has not been determined whether CsA regulates the IRFs or IFN, but our results showed no effect of CsA on IRF3 activation or on IFN-β production in HCECs.

The production of type I IFN is the first line of defense against viral infections, and it acts by limiting the early replication of viruses [19,20]. Deonarain et al. [21] demonstrated that IFN-β is crucial to this process because IFN-β-deficient mice were highly susceptible to viral infections. Our preliminary experiments showed that HSV infection was clearly depressed by poly(I:C) treatment prior to the HSV inoculation of the HCECs (data not shown). DEX treatment prior to HSV inoculation of HCECs led to the down-regulation of *TLR3* and *IFN-β* followed by increased *HSV-1* DNA and plaque formation. However, CsA did not interfere with the HSV-1 infection (data not shown). It is of interest to note that the anti-viral capabilities of corneal epithelial cells arise from their ability to produce IFN-β. Topical or systemic application of glucocorticoids results in the appearance of clinically active herpes keratitis, in which viral particles infect the corneal epithelial cells, leading to viral replication [9,10]. DEX has also been shown to increase the susceptibility of corneal epithelial cells to HSV-1 infection [44].

It has been known that TLR9 recognizes deoycytidylate-phosphate-deoxyguanosine (CpG) motifs in bacterial DNA, however, recent reports have demonstrated that TLR9 also recognizes CpG motifs in viral DNA, including HSV [24,45,46]. In addition, retinoic acid-inducible gene (RIG)-I-like receptors (RLRs), including RIG-I, melanoma differentiation-associated gene 5 (Mda5), and *Leishmania* G-protein 2 (LGP2), have recently been identified as cytoplasmic proteins that recognize viral RNA [47,48]. The RLRs also activate NFκB and IRF3 following viral infection and poly(I:C) stimulation. RLRs-mediated signaling induced by dsRNA has been demonstrated in epidermal keratinocytes [49]. Our results showed an elevated production of inflammatory cytokines/chemokines that was associated with an up-regulated expression of TLR3, indicating that TLR3/TRIF signaling pathways are involved in the anti-viral response of HCECs. However, the presence of signaling cannot be fully accounted for by the TLR3/TRIF signaling pathway alone. It is possible that the TLR9 and RLRs pathways may also play a role in the production of inflammatory cytokines/chemokines, but we did not study the RLRs pathway. Further investigation will be needed to determine the exact mechanisms.

In summary, we have demonstrated that HCECs have ability to produce inflammatory cytokines/chemokines via the innate immune system, and these responses can be modified by DEX and CsA. DEX down-regulated both NFκB and IRF3, whereas CsA down-regulated only NFκB. This inhibition by DEX of IRF3 followed by IFN-β production may be another mechanism in the immunosusceptibility of HCECs to HSV infection. Thus, the innate corneal immune system may be involved in HSV infection of HCECs, and further studies to determine the function of the innate immune system might lead to new therapeutic agents, or the development of effective ways of preventing corneal infections.
